# Fabrication and Characterization of Poly(hydroxybutyrate)- and Poly(caprolactone)-Based Active Biodegradable Films Incorporating Allyl Isothiocyanate

**DOI:** 10.3390/polym17091189

**Published:** 2025-04-27

**Authors:** Saliha Memis Karabuga, Perihan Kubra Akman, Fatih Tornuk

**Affiliations:** 1 Department of Food Engineering, Faculty of Chemical and Metallurgical Engineering, Yildiz Technical University, 34349 Istanbul, Türkiye; salihamemis92@gmail.com (S.M.K.); pkcicek@yildiz.edu.tr (P.K.A.); 2 Department of Nutrition and Dietetics, Faculty of Health Sciences, Sivas Cumhuriyet University, 58140 Sivas, Türkiye

**Keywords:** poly(hydroxybutyrate) (PHB), poly(caprolactone) (PCL), polymer blends, active biodegradable films, allyl isothiocyanate (AITC), solvent casting

## Abstract

In this study, in order to overcome the fragility and cost disadvantages of PHB-based films, PHB was blended with PCL. Additionally, allyl isothiocyanate (AITC) was incorporated as an active component. The resulting PHB, PCL, and PHB/PCL composite films with/without allyl isothiocyanate (AITC) prepared via the casting method were analyzed for their physicochemical, thermal, mechanical, barrier, morphological properties and antimicrobial and antioxidant activities. While neat PHB films showed the highest tensile strength (TS) of 19.82 MPa and the lowest elongation at break (EB) of 1.13%, PHB/PCL blend films exhibited lower TS (15.34 MPa) and higher EB values (21.33%). AITC addition decreased TS significantly while showing no significant impact on EB. PHB/PCL blend films had the highest water vapor permeability (WVP) values, possibly due to their increased porosity, while neat PCL- and PHB-based films showed better oxygen and water vapor barrier properties, respectively. DSC analysis showed that PHB and PCL films had a crystalline phase, while in the case of PHB/PCL blend films, both polymers maintained their characteristic melting behaviors. The addition of AITC affected the thermal stability by increasing the melting temperature of the PHB films and decreasing the melting temperature of the PCL films. SEM analyses revealed that PHB and PHB-A films had a homogeneous structure, while irregular spherical structures and cracks were also observed in PCL and PCL-A films. The incorporation of AITC into the film samples (PHB-A, PCL-A, and PHB/PCL-A) brought remarkable antimicrobial (from 16.25 mm to 37.25 mm of inhibition zones) and antioxidant activity (from 281.85 to 286.41 mg trolox equivalent/1 g film sample, as measured by CUPRAC), while no activity was observed in the control films without AITC (PHB, PCL, and PHB/PCL). In conclusion, new AITC-activated PHB-, PCL-, and PHB/PCL-based films were successfully designated with additional functionalities and showed valuable potential to be used in active biodegradable food packaging applications.

## 1. Introduction

The United Nations Environment Programme research estimates that over 300 million tons of plastic waste are produced annually worldwide, with just 9% of that material ever being recycled [1]. It is also speculated that plastic debris from 4 to 12 million metric tons enters the oceans each year [2]. Increasing awareness to reduce environmental damage caused by plastic waste, especially that produced by the food packaging industry, has led the food and packaging industry and the scientific community to work on the production of biodegradable and bio-based packaging materials [3].

Poly(3-hydroxybutyrate) (PHB) is one of the best-known members of the short-chain polyhydroxyalkanoates (PHAs) and is a biodegradable and renewable polymer composed of monomers with 3–5 carbon atoms. It is produced by several microorganisms intracellularly serving as a carbon reservoir. Biodegradable films produced from PHB have attracted great attention in recent years owing to several superiorities including being resistant to moisture and outstanding mechanical and barrier properties. PHB has similar mechanical properties with polypropylene (PP), which gives it the potential to be considered as a bio-based alternative [4]. However, it has various disadvantages such as its relatively high cost and brittleness, which limits its widespread use in food packaging. In order to overcome these disadvantages, various attempts have been made in recent years, such as blending PHB with other polymers to produce packaging materials with improved properties [5].

In previous studies, PHB has been blended with different polymers such as poly (ethylene oxide) (PEO), poly(L-lactide), poly (L-lactic acid) (PLLA), poly(D-lactide), PDLA, or poly(ε-caprolactone) (PCL) in order to produce packaging materials with improved properties. PCL is a biocompatible and biodegradable synthetic semi-crystalline linear polyester and can be readily degraded with enzymes produced by various microorganisms. Additionally, compared to PHB, it has a much lower melting temperature. It is ductile and can be blended with many polymers with different properties [6]. Moreover, PCL is an excellent co-polymer because of its high flexibility, heat stability, and biodegradability [5]. Considering all these features, it is quite advantageous to use PHB mixed with PCL to improve its properties as a packaging material. PCL improves the hardness and brittleness of PHB while at the same time increasing the flexibility of the material, allowing for wider applications [7,8,9].

The term “active packaging” refers to a type of packaging where the product, the environment, and the package work together to extend the shelf life of the product. A packaging material is turned into “active packaging” by using various bioactive substances such as antimicrobials, antioxidants, essential oil components, and herbal hydrosols, as well as CO_2_ emitters, oxygen, and ethylene scavengers, thus improving the quality characteristics of the food products in which it is used and extending their shelf life [10]. Among these bioactive substances, natural bioactives that have been commonly studied for food preservation include nisin, thymol, eugenol, carvacrol, AITC, and essential oil constituents. The U.S. Food and Drug Administration has classified these compounds as generally recognized as safe (GRAS). AITC is a lipophilic and colorless essential oil with a strong and pungent odor. It has strong antimicrobial effect, which can effectively inhibit the growth of various microorganisms [11]. It was originally extracted from plants such as horseradish, wasabi, mustard, and radish [12]. In this regard, the incorporation of packaging materials with AITC offers an effective solution for extending the shelf life of foods by preventing the growth of microorganisms thanks to its antimicrobial properties. Previous studies have demonstrated the potential use of AITC in various biodegradable polymer-based packaging systems including PLA, PLA-PCL, and cellulose acetate [12,13,14]. In this study, to the best of our knowledge, AITC was firstly incorporated into PHB-based films for antimicrobial and antioxidant purposes. Therefore, we aimed to design active packaging materials based on PHB, PCL, and PHB/PCL with added AITC and to determine the physicochemical, thermal, molecular, mechanical, barrier, morphological, antimicrobial, and antioxidant properties of these packaging materials. Unlike previous works, this research highlights the unique properties of PHB, such as its good biodegradability and mechanical strength, combined with the flexibility of PCL, to produce a novel packaging material. In addition, there is a need to develop innovative films by adding various natural bioactive components to biodegradable polymeric films to provide more effective and environmentally friendly solutions that improve food safety. Therefore, the active films produced in this study, with their improved physical and antimicrobial properties, may offer a promising approach in food packaging applications to eliminate both food safety and environmental concerns.

## 2. Materials and Methods

### 2.1. Materials

The PHB (poly-β-hydroxybutyrate) polymer PHB_R&D_ brand product (molecular weight (Mw) = 390,000–420,000 g/mol, particle size ≤ 300 µm) was obtained from Innovaplast Biotechnology Inc. (Eskişehir, Türkiye). Polycaprolactone (PCL) (average molecular weight (Mn) = 80,000 g/mol) was purchased from Merck (Darmstadt, Germany). Chloroform, which was used as a solvent to prepare the film solutions, was also provided by Merck (Darmstadt, Germany). Allyl isothiocyanate (AITC) was supplied by Sigma-Aldrich (St. Luis, MO, USA).

### 2.2. Film Preparation

**Figure 1:**
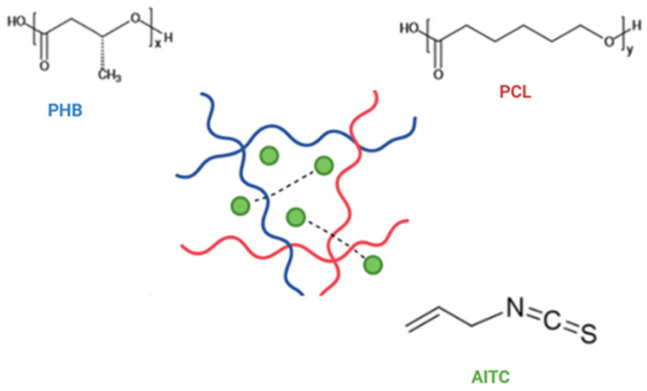
Schematic representation of the structural interaction between PHB, PCL, and AITC.

In this study, six different film samples were prepared using the solvent casting technique, and the formulations of the film-forming solutions were given in Table 1. The PHB/PCL ratio of the blend film was optimized based on preliminary trials, with higher PCL content selected for improved film structure. All the film solutions were prepared based on their target percent in chloroform (100 mL), which was used as the solvent. To obtain the homogeneity of the film-forming solutions, stirring was continued for an extra 10 min after the incorporation of AITC at the same conditions. The resulting homogeneous film solutions were carefully poured into glass Petri dishes (10 cm in diameter, with 30 mL of the film-forming solution per dish). Following drying the samples at room temperature for 8 h, the films were peeled off and kept at room temperature for 48 h in a desiccator for conditioning before testing. Structural interactions between PHB, PCL, and AITC are schematically shown in Figure 1.

### 2.3. Thickness

The thickness of the films was measured using a digital micrometer (Digimatic Indicator, Mitutoyo Corporation, Osaka, Japan). A minimum of six measurements were carried out at random locations on the films [15].

### 2.4. Moisture Content

The moisture content of the films was determined gravimetrically. For this purpose, the film sample (2 cm × 2 cm) was dried for 24 h at 105 °C in a drying oven, and the moisture percentage was calculated using Equation (1) [16].

(1) $$MC\left(\%\right)=\frac{m_{1}-m_{2}}{m_{1}}\times 100$$

m_1_: the initial sample weight; m_2_: the dried sample weight.

### 2.5. Optical Properties

A chromameter (CR- 400 Konica Minolta Sensing, Inc., Osaka, Japan) was used to measure the L*, a*, and b* values of the biodegradable films. The L*, a*, and b* values used in the color analysis represent the lightness/darkness (L*: 0 black; 100 white), redness/greenness (a*: positive values red; negative values green), and yellowness/blueness (b*: positive values yellow; negative values blue) axes, respectively. Following the calibration of the chromameter, the color measurement was carried out on the film samples placed on a white standard plate (L* = 93.49, a* = 0.25, b* = 0.09). At least five measurements were taken from various locations throughout the film [17].

### 2.6. Mechanical Properties

The mechanical properties of the biodegradable films were analyzed by using a texture analyzer (TA. XT Plus Stable Micro Systems, Surrey, UK) in accordance with the standard procedure of ASTM D882-12 [18]. Film samples were first cut into a rectangle form (1 × 14 cm) and then conditioned for 72 h at 43% relative humidity. The initial strain rate and grip distance were established at 1.25 cm/min and 10 cm, respectively. The program estimated the elongation at break (EB, %) and tensile strength (TS, MPa). Five repetitions were carried out for each film sample.

### 2.7. Barrier Properties

#### 2.7.1. Water Vapor Permeability

The water vapor permeability (WVP) of the biodegradable films was determined based on the protocol explained by Memiş et al. [19]. Prior to the analysis, glass cups containing silica gel were held at 105 °C for 24 h to eliminate any moisture; then, they were covered with film samples. After that, the covered cups were placed in desiccators with distilled water and kept at 25 °C for 24 h. During this period, the weight of the cups was recorded at the 0th, 5th, 15th, 20th, and 24th hours. The following formula was used to determine the WVP of each film (Equation (2)):

(2) $$WVP=\frac{W.X}{t.∆P.A}$$

where ΔP is the partial moisture pressure differential of the atmosphere between distilled water and the silica gel (2642 KPa at 24 °C), A is the area of the film samples in contact with the air (m^2^), W/t is the change in sample weight over time (g/h), and X is the film thickness (mm).

#### 2.7.2. Oxygen Permeability

The oxygen transmission rate (OTR) of the film samples was determined by measuring the peroxide value of antioxidant-free sunflower oil (15 mL) held in a conical flask (25 mL) covered with the film samples, based on the method by Kurt and Kahyaoglu with slight modifications [20]. The peroxide value of the oil samples was analyzed using the sodium thiosulfate titration technique after keeping the oil-containing flasks in dark conditions at 25 °C for 9 days.

### 2.8. Thermal Characteristics

DSC analysis was carried out to reveal the thermal properties of the films using DSC equipment (DSC Q20, TA Instruments, Inc., New Castle, DE, USA). Approximately 5–8 mg of each film sample was sealed in an aluminum pan and heated from 30 °C to 200 °C at a rate of 10 °C/min. The DSC thermograms formed were used to calculate the melting point (Tm) and melting enthalpy (H) of the samples. An empty aluminum pan was used as a reference [18].

### 2.9. SEM

The SEM images of the film samples were obtained using a Zeiss EVO LS 10 scanning electron microscope at 5 kV working voltage with 10,000× magnification under vacuum conditions. Film samples were coated with gold prior to analysis [15].

### 2.10. FTIR

The film samples were molecularly characterized using an FTIR spectrophotometer (Bruker Tensor 27, Bremen, Germany). The measurements were conducted at the wavelength range of 4000–400 cm^−1^ at room temperature [19].

### 2.11. Film Biodegradability

To determine the biodegradability of the films, a 2 cm^2^ piece of each sample was dried in a desiccator until constant weight (starting weight). The samples were then buried in 100 g of soil, and the daily weight changes were recorded for 60 days. Each sample was then dried to a fixed weight (final weight) [21]. The percentage weight loss (W%) was calculated using the following Equation (3):

(3) $$W\left(\%\right)=\left[\frac{initial\;weight-final\;weight}{initial\;weight}\right]\times 100$$

Biodegradability tests were conducted using at least three parallel samples for each film group, and the results were reported based on the average values. The complete biodegradation time for each film was estimated by extrapolating the 60-day weight loss data using logarithmic trendline equations (R^2^ > 0.95) in Microsoft Excel for Microsoft 365 (Microsoft Corporation, Redmond, WA, USA).

### 2.12. Antimicrobial Activity

The antibacterial activities of the film samples were evaluated against Gram-positive strains of *Staphylococcus aureus* (ATCC 25923), *Bacillus cereus* (FMC19), and *Listeria monocytogenes* (ATCC 13932) and Gram-negative strains of Escherichia coli O157:H7 (ATCC 25922) and *Salmonella enterica* subsp. *enterica* serovar Typhimurium (ATCC 14028) using the agar diffusion method [22]. Firstly, cryopreserved strains were activated twice for 24 h at 37 °C in nutrient broth (Merck, Germany). The films were cut into square pieces (1 × 1 cm^2^) and then arranged on nutrient agar (Merck, Germany)-containing Petri plates that were previously inoculated with 1% (v:v) of the activated strains’ broth culture.

Then, the Petri plates were incubated for 24 h at 37 °C, and the clear (inhibition) zones that developed around the films were measured. The results were recorded in mm.

### 2.13. Antioxidant Activity

The antioxidant capacity of the biodegradable films was determined by making some modifications to the CUPRAC method developed by Apak et al. [23]. For this purpose, 100 mg of the film sample was mixed with 10 mL of the extraction solvent (80:20 methanol/water *v*/*v*) and sonicated for 3 min. The extracts were filtered using a 0.45 μm filter. To determine the antioxidant capacity, 0.1 mL of extract was mixed with 1 mL of the CuCl_2_ solution (170.48 mg CuCl_2_.2H_2_O/100 mL water), 1 mL of the Nc solution (0.156 g Nc/100 mL ethanol) and 1 mL of the NH_4_Ac solution (7.708 g NH_4_Ac/100 mL water); then, 1 mL of distilled water was added to the mixture to complete the volume to 4.1 mL. The mixture was incubated at room temperature for 60 min. Absorbance was measured at 450 nm. Antioxidant capacity results were expressed as mg Trolox equivalent per 1 g film sample (mg TE/1 g FS).

### 2.14. Statistical Analysis

The analyses were carried out in triplicate. A Windows-based statistical analysis software (SAS 8.2, SAS Institute, Cary, NC, USA) was used to perform the statistical analyses. Tukey’s test was used to assess the statistical differences between the means at the 95% significance level after a one-way analysis of variance (ANOVA) was carried out.

## 3. Results and Discussion

### 3.1. Thickness

Film thickness is a critical criterion which directly affects the gas permeability and mechanical properties of the films. The thickness values of the film samples are given in Table 2. As seen in the table, the thickness of the film samples varied between 0.17 mm and 0.299 mm. The addition of AITC to neat PHB and PCL films did not cause a significant difference (*p* > 0.05) in the film thicknesses, whereas a statistically significant difference was observed between the PHB/PCL and PHB/PCL-A films (*p* < 0.05). Similar results were observed in studies where the thickness increased with the incorporation of an essential oil into the active packaging film. This may be due to the distribution of the essential oil in the film matrix [24].

### 3.2. Moisture Content

The moisture contents of the film samples are given in Table 2 and varied between 0.270% and 0.215%. The addition of AITC to the film matrix decreased the moisture content of the films, but this decrease was not always statistically significant (*p* > 0.05). This may be due to the hydrophobic nature of AITC. Similar findings have been reported in the literature, and it has been shown that hydrophobic additives reduce the moisture level of the film [16]. In particular, the moisture content of PHB-A films (0.215 ± 0.008%) was found to be lower than that of PHB films (0.254 ± 0.017%), and similarly, PCL-A films (0.218 ± 0.003%) showed lower moisture content than that of PCL films (0.270 ± 0.027%). This trend suggests that the presence of AITC in the film matrix reduces the moisture content as a result of its interaction with the polymer structure. These findings are consistent with previous studies showing that hydrophobic components can reduce the water content of biodegradable films. For example, Mutlu reported that the addition of grape seed oil (GSO) to gelatin/guar gum films reduced the moisture content from 17.52% to 15.01%, which they attributed to hydrophobic interactions between the oil and the polymer matrix [16]. Similarly, Nisar et al. determined that clove bud essential oil (CEO) reduced the moisture content of pectin films from 18.43% to 10.14%, supporting the role of hydrophobic additives in enhancing water resistance [25].

### 3.3. Color

The optical properties of packaging materials play an essential role on product appearance and consumer preferences. The color parameters of the films are shown in Table 2. With the addition of AITC, significant (*p* < 0.05) changes occurred in the color values of the film samples. Considering the L* values, the addition of AITC caused a significant decrease only in the PHB and PHB-A films (*p* < 0.05). With the addition of AITC, slight but significant (*p* < 0.05) changes were observed in the a* and b* values of the film samples. The difference in a* and b* values can be caused by the amber hue of the active compounds added into the films [18]. Many previous studies have reported that adding various oils to biodegradable films caused significant color changes [16,18,24].

### 3.4. Mechanical Properties

Mechanical performance in tensile strength and elongation at break for the prepared biodegradable films are shown in Table 3. The TS values of the PHB, PCL, and PHB/PCL films were found to be 19.82, 12.21, and 15.34 MPa, respectively. With the addition of AITC, the TS values of these films showed a decreasing trend to 17.92, 10.38, and 13.88 MPa, respectively. The principal element that may explain the decrease in TS in the polymer/AITC composites is the partial substitution of weaker polymer–oil interactions for stronger polymer–polymer interactions [18]. The EB values of the PHB, PCL, and PHB/PCL films were found to be 1.13, 53, and 21.67 (%). It was found that the EB value of the PHB-PCL mixture was higher than that of PHB. It is observed that the hard structure of PHB is significantly improved by the soft and flexible structure of PCL [8]. With the addition of AITC, the EB values of these films showed an increasing trend to 1.27, 55.67, and 21.67 (%). These increases in the EB values of the films show the plasticizing effect of adding AITC to the films. The PHB and PHB-A films showed higher TS and lower elongation at break than other films, indicating that they are hard and brittle [26]. TS refers to the maximum applied stress that a film can withstand while being stretched, while EB relates to the stretchability of films before breaking. The results were found to be compatible with the studies conducted by Sharma et al. [10]. The addition of AITC was mostly localized between the polymeric chains of the films, weakening the intermolecular forces holding adjacent chains. Therefore, it facilitated the movements of the polymeric chains, resulting in an increase in breaking elongation and the stretching ability of the films. The TS value of the PHB/PCL film was found to be significantly lower than the TS value of the PHB film and significantly higher than the TS value of the PCL film. The TS values of the PHB/PCL film were found to be compatible with the studies conducted by Garcia and Przybysz [6,27]. The immiscibility between biopolymers affects the mechanical properties of the blends; i.e., the individual mechanical properties of homopolymers are not necessarily transferred to the blends [28]. An inverse relationship was observed between the tensile strength (TS) and elongation at break (EB) of all films. Strong intermolecular forces lead to higher tensile strength because the forces required to break these bonds are greater. On the other hand, strong forces limit the stretch ability of the films significantly before failure, leading to a lower EB value [29].

### 3.5. Barrier Properties

WVP is an important parameter since it indicates how much water vapor permeates per unit area and time via the packaging material. No significant difference was found between the WVP values of the PHB and PHB-A and PHB/PCL and PHB/PCL-A films, as seen in Table 3. The addition of AITC to the PCL film caused a significant increase in the WVP value of the PCL-A film. The increase in the WVP of the film can be attributed to the increase in the porosity and intermolecular mobility of the polymer chains. A significant increase in the porosity of the PHB/PCL and PHB/PCL-A films was observed in the SEM images, which increased the water vapor permeability (WVP) values of the films to 0.212 and 0.174 g mm/m^2^ h kPa, respectively. It has been reported that the increase in the water vapor permeability (WVP) of the films is associated with the increase in their porosity, and the results obtained are consistent with the study conducted by Sharma et al. [10].

The transmission of oxygen through packaging films accelerates the deterioration process of food and negatively affects product quality. Therefore, an ideal packaging film should have low permeability to the surrounding atmosphere [30]. The peroxide values (PVs) of sunflower oil protected with the films are shown in Table 3. The PV value of the neat PHB film was found to be 23.05, while the addition of AITC into the polymer matrix caused the PV value to increase to 27.08 depending on the increase in the oxygen permeability of the PHB-A film. PHAs exhibit relatively strong water vapor and moderate oxygen barrier properties. Various semi-crystalline regions, such as the mesomorphic and melt regions in PHB, are permeable to oxygen molecules and water vapor [31]. On the other hand, PCL-based films showed lower (*p* < 0.05) PVs, indicating better oxygen barrier properties. This result is consistent with the study by Arrieta and López et al. [32]. The PVs (22.70 and 21.69 meq/kg) of the PHB/PCL and PHB/PCL-A films were between those of the PHB and PCL films, indicating that the blend partially preserved the barrier properties of both polymers.

### 3.6. Thermal Characteristics

**Figure 2:**
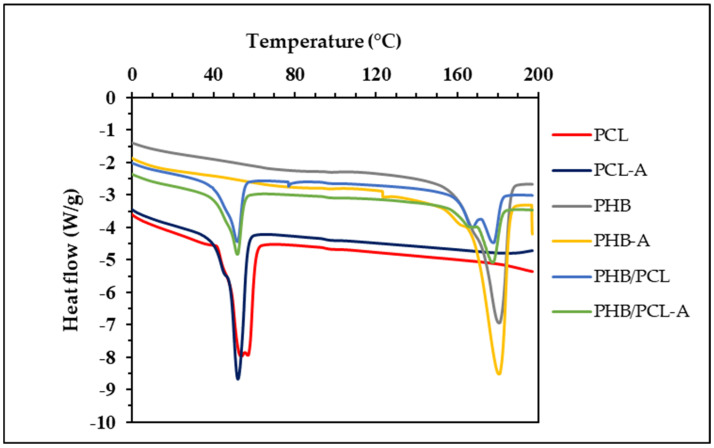
DSC thermograms of the films.

The thermal properties of the film samples are presented in Figure 2 and Table 4. The T_onset_, T_peak_, and T_end_ values in the table show the temperatures at which the melting process starts, is fastest, and ends, respectively. In the thermograms of the PHB and PCL films, one endothermic peak was observed in each film sample, indicating that both films have a crystalline phase [33]. The PHB film showed a single melting endotherm at 180.27 °C (ΔH = 89.69 J/g), while the PCL film melted at 53.71 °C (ΔH = 50.35 J/g), confirming their semi-crystalline nature. However, two endothermic peaks were observed in the thermogram of the PHB/PCL blend film at 177.67 °C and 51.79 °C, which correspond to PHB and PCL, respectively. This indicates that the blending of these polymers did not affect their thermal properties, and they retained their characteristic melting behaviors [8]. The melting temperature of PHB showed minimal change (180.27 °C to 180.69 °C) after AITC incorporation, suggesting that AITC had little effect on PHB’s crystalline structure. Moreover, the decrease in ΔH2 value following AITC incorporation (89.69 J/g to 69.70 J/g) suggests reduced crystallinity in PHB, potentially due to disrupted polymer chain organization during crystallization. This observation is consistent with reported plasticizing effects of essential oil components in biopolymeric matrices [34]. The incorporation of AITC into the PCL film caused a decrease in the melting temperature of the PCL-A film, indicating that the PCL-A film had lower thermal stability than the PCL film.

### 3.7. SEM

**Figure 3:**
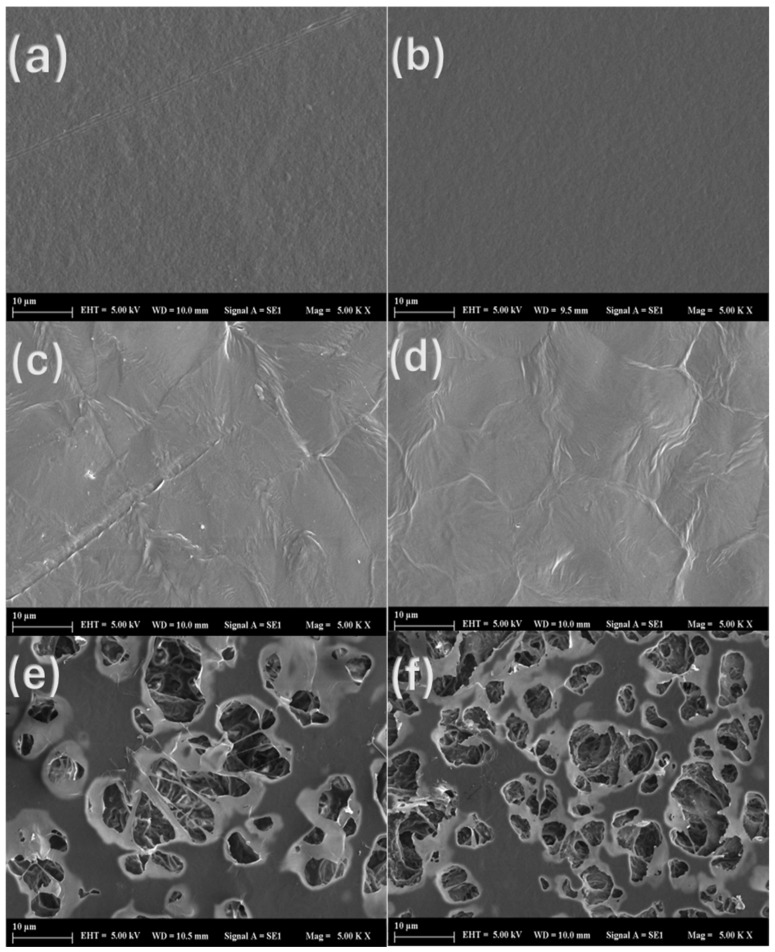
SEM micrographs of (**a**) the PHB film, (**b**) the PHB-A film, (**c**) the PCL film, (**d**) the PCL-A film, (**e**) the PHB/PCL film, and (**f**) the PHB/PCL-A film.

The micrographs of the films obtained through SEM analysis are shown in Figure 3. The PHB (a) and PHB-A (b) film samples showed a clean and homogenous microstructure with no cracks or contaminants. According to the findings by Sharma et al., the creation of a few pores in the PHB (a) and PHB-A (b) micrographs was caused by chloroform evaporation from the films during the drying process [24]. The structure of less regular spherical hexagons with few cracks, characteristic for some polymer types, was also observed in PCL (c) and PCL-A (d) films [35]. In the micrographs of PHB/PCL (e) and PHB/PCL-A (f) films, the low miscibility of the polymers and the limited chemical interaction were clearly seen, as seen by the two-phase structure. A similar immiscibility of both polymers was also reported by Garcia-Garcia et al. [8]. However, a nearly homogeneous dispersion of PCL droplets in PHB was also observed in Figure 3e,f. Indeed, the mechanical properties of blend films are closely related to the molecular interactions and structural integrity of individual polymers and additional additives. Therefore, as seen in Table 3, increasing elongation and decreasing the tensile properties of blend films as compared to neat PHB indicate that the desired goals were achieved.

### 3.8. FTIR

**Figure 4:**
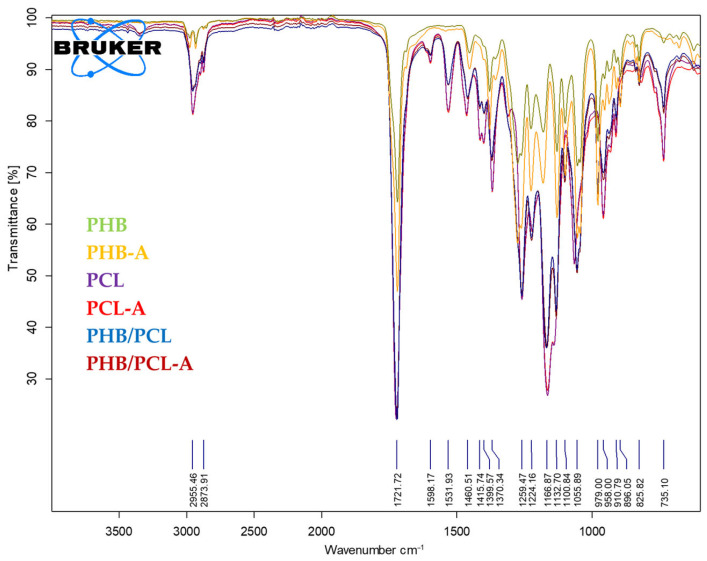
FTIR spectrum of the films.

The FTIR spectra of the films are presented in Figure 4 at the wavelength range of 4000–400 cm^−1^. The PHB/PCL spectrum showed characteristic bands of both PHB and PCL. The addition of AITC did not make considerable changes in the FTIR spectra of the films, indicating that no new chemical bonds were formed between the film constituents, and AITC was physically embedded in the polymer matrix. The broad band at 3000–2900 cm^−1^ is attributed to singlet -CH_3_, asymmetric CH_2_-, and -CH_2_- symmetric stretching vibrations; the characteristic peak observed at 1722 cm^−1^ for all samples is associated with ester carbonyl C=O found in PCL and PHB. The peak at 1280 cm^−1^ is attributed to C-O stretching; The peak around 1165 cm^−1^ is attributed to C-O-C stretching [6].

New peaks were observed at 1644 cm^−1^ and 3395 cm^−1^. The PCL/PHB mixture showed the identification of C=C bands and the -OH group, respectively. It is well known that thermal degradation of PHB occurs close to its melting point, leading to random chain scission. This result confirms the degradation of PHB under the PCL/PHB mixture preparation conditions [27].

### 3.9. Film Biodegradability

**Figure 5:**
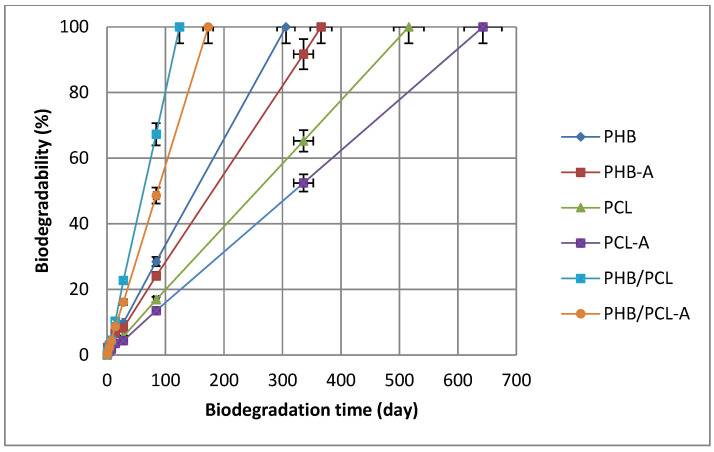
Biodegradability in the soil of films.

The results obtained in the soil burial degradation experiment of the films are shown in Figure 5. The PHB/PCL and PHB/PCL-A films showed faster degradation, while lower degradation rate was observed in the neat PCL and PCL-A films. The remarkable degradation rate of the blend films may be due to their large surface area, high porosity level, and three-dimensional structure, which allow for more mass of microorganisms to attach to the polymeric films [36]. PHB and PHB-A films degraded faster than PCL and PCL-A films. The advantage of PHB is that its degradation takes place in both aerobic and anaerobic conditions, resulting in a fast degradation rate. PHB-A, PCL-A and PHB/PCL-A films appear to degrade later than the others. Slower degradation of AITC-incorporated films can be attributed to AITC’s inhibition effect on the microbial population that causes degradation [26]. Our results were consistent with the findings about the biodegradation behavior of PCL/PHB blends reported by La Cara et al. [37]. Factors such as soil temperature, nutrients, pH, oxygen, and salinity affect the survival and activity of microorganisms. Differences in the literature on the degradation of films can be attributed to the different composting conditions adopted [38].

### 3.10. Antimicrobial Activity

The antimicrobial activities of the films against foodborne pathogenic bacteria were determined, and the results are shown in Table 5. As expected, AITC-free films showed negligible antibacterial activity against test microorganisms. This was consistent with previously reported studies. For example, in a study where cinnamon, melaleuca, and citronella essential oils were incorporated in PHB-based films, it was reported that pure PHB did not exhibit antimicrobial activity [26]. Similarly, a study on PHB films containing different amounts of canola oil reported that pure PHB in the control group had no antimicrobial effect [39]. Furthermore, neat PCL-based films did not show any inhibitory activity on Salmonella and Listeria [40]. In this study, AITC-containing films (PHB-A, PCL-A, and PHB/PCL-A) showed high antimicrobial activity against all bacteria. Specifically, the PHB-A film showed the highest inhibitory effect on *E. coli* O157:H7 (37.25 ± 5.31) and *S.* Typhimurium (31.50 ± 4.93), while the PCL-A film also recorded significant inhibitory effects on *E. coli* O157:H7 (33.25 ± 3.86) and *S.* Typhimurium (36.25 ± 4.03). The PHB/PCL-A film showed lower activity compared to other AITC films but still showed antimicrobial activity against all bacteria. The lower antimicrobial activity of PHB/PCL-A films may be due to factors such as the structure of the film matrix, AITC distribution, release kinetics, surface properties, and inter-polymer interactions. Studies support that AITC has strong inhibitory effects on both Gram-positive and Gram-negative pathogenic bacteria, while Gram-negative bacteria are more sensitive to AITC inhibition than Gram-positive bacteria [41]. In a study examining the antimicrobial effect of PLA-based films containing AITC (allyl isothiocyanate), the findings showed that these films significantly inhibited the growth of pathogens such as *E. coli* and *L. innocua* in grape and ready-made turkey meat samples [12]. The study revealed that AITC was effective against foodborne bacteria and could be used as an active agent in the biodegradable active packaging material. In a study examining the bactericidal effects of AITC on *E. coli* O157:H7, *S.* Typhimurium, and *L. monocytogenes*, it was reported that the most sensitive and resistant species to AITC was *E. coli* O157:H7 and *L. monocytogenes*, respectively. AITC exhibits its antimicrobial effects on microorganisms by disrupting the cell membrane, increasing permeability, causing the leakage of intracellular metabolites, inhibiting DNA and protein synthesis, and creating oxidative stress by producing reactive oxygen species (ROS) [42]. The findings are consistent with the results of our study.

### 3.11. Antioxidant Activity

The antioxidant capacities of the film samples were measured using the CUPRAC method and were found to be approximately 280–286 mg TE/1 g FS (Trolox Equivalent/1 g Film Sample) (Table 5). Neat PHB, PCL, and PHB/PCL films did not show any measurable antioxidant activity, while the addition of AITC brought them a strong antioxidant effect, which indicated that antioxidant activity originated from AITC. It was also clear from the table that the differences between the antioxidant activity of PHB-A, PCL-A, and PHB/PCL-A were insignificant (*p* > 0.05). The antioxidant effect of AITC and AITC-incorporated films have been specified in the literature [14,23,43]. Studies have shown that AITC not only has an antimicrobial effect but also a protective effect against oxidative damage; it achieves this effect by activating the Nrf2/ARE pathway, one of the cell defense mechanisms under oxidative stress. Under oxidative stress conditions, AITC ensures that the Nrf2 transcription factor is separated from the Keap1 protein; the released Nrf2 is transported to the nucleus, binds to ARE (antioxidant response element) regions, and increases the expression of phase II detoxification enzymes such as HO-1, NQO1, and GST. These enzymes reduce cellular damage by neutralizing reactive oxygen species (ROS) and contribute to the removal of toxic compounds from the body. Therefore, AITC stands out as an important antioxidant agent that strengthens cellular defense systems [44].

Essential oils have antioxidant properties in addition to providing antimicrobial effects [18]. It is thought that the strong antioxidant activity of AITC can prevent or delay oxidation reactions in various foods. These findings are consistent with other studies in the literature. For example, it has been reported that antioxidant activity significantly increases with increasing CEO concentration in citrus pectin films enriched with clove bud essential oil (CEO). The lowest total phenolic content (0.49 mg gallic acid/g) was observed in the pure pectin film (control), while the highest value (13.52 mg gallic acid/g) was recorded in the film containing 1.5% CEO [25]. Similarly, in chitosan films combined with summer thyme (*Satureja hortensis* L.) essential oil (SHEO), antioxidant activity was reported to increase up to 66.08% as the SHEO concentration increased. No DPPH radical scavenging activity was observed in the control film without SHEO [22]. These studies support the effect of essential oils in increasing the antioxidant capacity of films.

## 4. Conclusions

This study successfully demonstrated the potential of developing biodegradable and bioactive films based on PHB, PCL, PHB/PCL, and their blends incorporated with AITC. The incorporation of AITC significantly enhanced the antimicrobial properties of the films, while the blending of PHB with PCL improved their mechanical performance. These findings highlight the promising potential of these novel materials as sustainable alternatives to conventional plastic packaging. However, further research is warranted to optimize film formulations, evaluate their long-term stability, and assess their interactions with different food products under real-world conditions. Future studies should also focus on scaling up production and conducting comprehensive life cycle assessments to evaluate the environmental impact of these films.

## Figures and Tables

**Table 1:** Preparation details of biodegradable film solutions (film-forming solutions were prepared based on 100 mL of chloroform as the solvent).

Film Code	Polymer Composition	Polymer (g)	AITC (mL)	Key Process Parameters
PHB	PHB	2.00	0	55 °C, 7–8 h stirring
PHB-A	PHB + AITC	2.00	1	55 °C, 7–8 h + 10 min AITC
PCL	PCL	4.00	0	RT, 20 min stirring
PCL-A	PCL + AITC	4.00	1	RT, 20 min + 10 min AITC
PHB/PCL	PHB:PCL (1:2)	1.00 PHB + 2.00 PCL	0	55 °C (PHB) + RT (PCL) mix
PHB/PCL-A	PHB:PCL (1:2) + AITC	1.00 PHB + 2.00 PCL	1	55 °C (PHB) + RT (PCL) + 10 min AITC

RT: room temperature.

**Table 2:** Thickness, percent moisture, and optical properties of the films.

Film Samples	Thickness (mm)	Moisture Content (%)	L*	a*	b*
PHB	0.183 ± 0.007 ^d^	0.254 ± 0.017 ^ab^	90.50 ± 0.33 ^b^	0.14 ± 0.02 ^b^	1.60 ± 0.29 ^d^
PHB-A	0.171 ± 0.009 ^d^	0.215 ± 0.008 ^b^	89.12 ± 0.20 ^c^	0.20 ± 0.08 ^b^	2.13 ± 0.38 ^c^
PCL	0.210 ± 0.014 ^c^	0.270 ± 0.027 ^a^	90.18 ± 0.32 ^b^	0.41 ± 0.03 ^a^	0.23 ± 0.05 ^e^
PCL-A	0.222 ± 0.010 ^c^	0.218 ± 0.003 ^b^	90.24 ± 0.25 ^b^	0.17 ± 0.04 ^c^	2.52 ± 0.09 ^b^
PHB-PCL	0.267 ± 0.025 ^b^	0.256 ± 0.010 ^ab^	95.16 ± 0.46 ^a^	0.52 ± 0.02 ^d^	2.12 ± 0.04 ^c^
PHB-PCL-A	0.299 ± 0.016 ^a^	0.232 ± 0.020 ^ab^	95.83 ± 0.64 ^a^	0.81 ± 0.04 ^e^	3.48 ± 0.15 ^a^

Note: different letters in the same column indicate significant differences between the results (*p* < 0.05).

**Table 3:** Mechanical and barrier properties of the PHB, PCL, and PHB/PCL films activated with AITC.

Film Samples	Tensile Strength (MPa)	Elongation at Break (%)	WVP (g mm h^−1^ m^−2^ kPa^−1^)	PV (meq/kg)
PHB	19.82 ± 2.86 ^a^	1.13 ± 0.37 ^c^	0.041 ± 0.009 ^b^	23.05 ± 3.38 ^ab^
PHB-A	17.92 ± 0.18 ^ab^	1.27 ± 0.64 ^c^	0.027 ± 0.004 ^b^	27.08 ± 1.71 ^a^
PCL	12.21 ± 2.17 ^bc^	53.00 ± 7.55 ^a^	0.056 ± 0.010 ^b^	18.53 ± 1.73 ^b^
PCL-A	10.38 ± 2.32 ^c^	55.67 ± 8.02 ^a^	0.192 ± 0.010 ^a^	18.48 ± 1.45 ^b^
PHB-PCL	15.34 ± 2.25 ^abc^	21.33 ± 7.51 ^b^	0.212 ± 0.046 ^a^	22.70 ± 1.75 ^ab^
PHB-PCL-A	13.88 ± 1.86 ^bc^	21.67 ± 7.09 ^b^	0.174 ± 0.013 ^a^	21.69 ± 1.10 ^ab^

Note: different letters in the same column indicate significant differences between the results (*p* < 0.05).

**Table 4:** DSC values of the PHB, PCL, and PHB/PCL films activated with AITC.

Film Samples	T1_onset_ (°C)	T1_peak_ (°C)	T1_end_ (°C)	ΔH1 (J/g)	T2_onset_ (°C)	T2_peak_ (°C)	T2_end_ (°C)	ΔH2 (J/g)
PHB	-	-	-	-	142.29	180.27	192.35	89.69
PHB-A	-	-	-	-	161.51	180.69	190.56	69.70
PCL	40.16	53.71	66.53	50.35	-	-	-	-
PCL-A	18.93	51.94	62.51	54.93	-	-	-	-
PHB/PCL	27.65	51.79	60.50	24.53	151.45	177.67	185.42	27.94
PHB/PCL-A	33.01	51.84	61.17	22.05	154.58	177.05	182.42	26.26

**Table 5:** Antimicrobial (inhibition zones, mm) and antioxidant activities of PHB, PCL, and PHB/PCL films activated with AITC.

Film Sample	*S. aureus*	*B. cereus*	*L.* *monocytogenes*	*E. coli* O157:H7	*S.* Typhimurium	Antioxidant Capacity CUPRAC (mg TE/1 g FS)
PHB	00.00 ± 0.00 ^Ba^	00.00 ± 0.00 ^Ca^	00.00 ± 0.00 ^Da^	00.00 ± 0.00 ^Ca^	00.00 ± 0.00 ^Ca^	-
PHB-A	30.75 ± 3.30 ^Ab^	30.50 ± 2.08 ^Ab^	20.75 ± 2.06 ^Bc^	37.25 ± 5.31 ^Aa^	31.50 ± 4.93 ^ABb^	286.41 ± 4.75 ^A^
PCL	00.00 ± 0.00 ^Ba^	00.00 ± 0.00 ^Ca^	00.00 ± 0.00 ^Da^	00.00 ± 0.00 ^Ca^	00.00 ± 0.00 ^Ca^	-
PCL-A	32.50 ± 3.00 ^Aab^	30.00 ± 4.24 ^Abc^	24.50 ± 2.88 ^Ac^	33.25 ± 3.86 ^Aab^	36.25 ± 4.03 ^Aa^	283.10 ± 6.22 ^A^
PHB/PCL	00.00 ± 0.00 ^Ba^	00.00 ± 0.00 ^Ca^	00.00 ± 0.00 ^Da^	00.00 ± 0.00 ^Ca^	00.00 ± 0.00 ^Ca^	-
PHB/PCL-A	29.50 ± 4.65 ^Aa^	18.25 ± 2.75 ^Bb^	16.25 ± 1.50 ^Cb^	26.00 ± 1.83 ^Ba^	25.50 ± 1.91 ^Ba^	281.85 ± 4.68 ^A^

Note: different uppercase letters in the same column indicate significant difference (*p* < 0.05) between the results, while different lowercase letters within the same line show that the results are significantly different (*p* < 0.05).

## Data Availability

The original contributions presented in this study are included in the article material. Further inquiries can be directed to the corresponding author.
